# Concordance of Corneal Tomographic Measurements Obtained via Swept-Source Optical Coherence Tomography Versus Scheimpflug Tomography

**DOI:** 10.3390/bioengineering13080944

**Published:** 2026-08-21

**Authors:** Kamini Narendra Reddy, Ramadhan Ahmed, Matthew Zeng, Nakul S. Shekhawat

**Affiliations:** 1 Wilmer Eye Institute, Johns Hopkins University School of Medicine, Baltimore, MD 21287, USA; kreddy13@jh.edu; 2 School of Medicine, Johns Hopkins University School of Medicine, Baltimore, MD 21205, USA; rahmed21@jh.edu; 3 Department of Biology, Krieger School of Arts and Sciences, Johns Hopkins University, Baltimore, MD 21218, USA; xzeng27@jhu.edu

**Keywords:** cornea, pachymetry, swept source optical coherence tomography, Scheimpflug tomography

## Abstract

Purpose: To compare concordance of corneal tomographic measurements obtained using Anterion versus Pentacam HR. Setting: Outpatient ophthalmology clinic. Design: Retrospective cross-sectional study. Methods: Eyes without corneal abnormalities underwent tomography using the Anterion Cornea App and Pentacam HR. Measurements included anterior keratometry (K1, K2, ΔK, average K, K_max_), posterior keratometry (K1, K2, ΔK, and average K), astigmatism power-vector components (J0, J45) for both surfaces, central corneal thickness (CCT), white-to-white (WTW), and thinnest pachymetric point (TP). We assessed agreement using Pearson correlation coefficients (ρ), Lin’s concordance correlation coefficient (CCC), and Bland–Altman analysis. Generalized estimating equation models were used to assess measurement differences between devices. Results: A total of 64 eyes of 41 patients underwent corneal tomography with both devices. Anterion and Pentacam HR showed strong linear correlations for all measurements (ρ > 0.700), with concordance ranging from satisfactory to excellent (CCC 0.704–0.981). No statistically significant mean inter-device differences were found for anterior K1 (0.01 diopters [D], *p* = 0.775), K2 (−0.003 D, *p* = 0.951), average K (0.01 D, *p* = 0.801), ΔK (−0.02 D, *p* = 0.693), or the anterior astigmatism vectors J0 (0.002 D, *p* = 0.936) and J45 (0.03 D, *p* = 0.129). Mean posterior K1, K2, and average K measurements were flatter with Anterion than Pentacam HR, with mean inter-device differences of 0.13 D (*p* < 0.001), 0.11 D (*p* < 0.001), and 0.12 D (*p* < 0.001), respectively. The mean inter-device difference for posterior ΔK was −0.02 D (*p* = 0.018). Statistically significant mean inter-device differences were also found for posterior J0 (−0.01 D, *p* = 0.018) and J45 (0.01 D, *p* = 0.007). Anterion measured lower values than Pentacam HR for anterior Kmax, CCT, and TP, with mean inter-device differences of −0.24 D (*p* = 0.002), −7.66 μm (*p* < 0.001), and −8.58 μm (*p* < 0.001), respectively, and a higher mean WTW, with a mean inter-device difference of 0.07 mm (*p* < 0.001). Conclusions: Anterion and Pentacam HR showed satisfactory-to-excellent measurement concordance, with negligible mean inter-device biases for most anterior keratometry measurements and anterior astigmatism vectors. However, individual-eye differences could approach 0.49–0.61 D for anterior keratometry and exceed 20 μm for pachymetry. These differences may become relevant in borderline cases and during longitudinal disease monitoring, warranting parameter-specific caution.

## 1. Introduction

Corneal tomography, which measures curvature of the front and back of the cornea as well as corneal thickness, is an essential part of cataract, corneal, and refractive surgery planning. Accurate tomographic measurements are essential for optimizing intraocular lens (IOL) selection to achieve desired refractive outcomes in cataract surgery, identifying eligible candidates for laser refractive surgery, and screening for corneal ectasia. The Oculus Pentacam HR is widely used for tomography in the United States (US) and uses a rotating Scheimpflug camera to provide a 3D reconstruction of the cornea and anterior chamber of the eye [1]. The Heidelberg Anterion uses swept-source optical coherence tomography (SS-OCT) technology with a 1300 nm wavelength to improve image acquisition [2]. The Anterion’s Cornea App obtains 65 radial B-scans to provide high-resolution cross-sectional images of the entire anterior chamber of the eye and precise anterior and posterior corneal biometry [2]. Scheimpflug tomography and SS-OCT use fundamentally different imaging principles to characterize the cornea. Scheimpflug tomography reconstructs the anterior segment using rotating blue-light imaging (475 nm), whereas SS-OCT uses a longer wavelength near-infrared light source (1300 nm) to generate high-resolution cross-sectional images of the cornea [2]. These technical differences may influence the measurement of corneal parameters and contribute to differences in agreement between devices.

The Heidelberg Anterion platform received US Food and Drug Administration (FDA) clearance for clinical use in the United States in October 2023. Few prior studies have compared corneal tomographic measurements obtained with the Pentacam and Anterion, and these were conducted in single, non-US centers [3,4,5]. These studies also varied in the parameters they assessed and in their analytic approaches and reached differing conclusions regarding the agreement and interchangeability of the two devices. For example, Ang et al. [3] reported good agreement in anterior keratometry values, whereas Gim et al. [4] found that most keratometry values could not be used interchangeably. This lack of consensus has left the measurement agreement and interchangeability of Anterion and Pentacam unresolved for corneal tomography.

The purpose of this study was to comprehensively evaluate the concordance of corneal tomographic measurements, including anterior and posterior keratometry, central corneal thickness, thinnest pachymetric point, and white-to-white diameter obtained using Anterion versus Pentacam HR in a US clinical population.

## 2. Methods

### 2.1. Study Population

This was a retrospective cross-sectional study of patients presenting to the Wilmer Eye Institute, Johns Hopkins Medicine, in Baltimore and Bethesda, Maryland. Patients were seen from August 2024 to February 2025. The study included eyes with healthy corneas and no history of corneal surgery. Corneal status was ascertained by chart review of the documented clinical diagnosis and slit-lamp examination findings recorded by the treating ophthalmologist. Specific tomographic indices (e.g., the Belin/Ambrósio Enhanced Ectasia Display or keratoconus indices) were not used to define corneal normality or to exclude eyes. We excluded eyes that had a prior diagnosis of keratoconus, prior refractive surgery, Fuchs endothelial dystrophy, corneal edema, pterygium, anterior basement membrane dystrophy, Salzmann nodular degeneration, corneal scarring, or moderate-to-severe ocular surface disease such as dry eye. Eyes with cataract or a remote history of uncomplicated cataract surgery without any corneal abnormalities remained eligible. As this was a retrospective study, no a priori sample size calculation was performed. The final sample of 64 eyes is comparable to prior device-concordance studies of the Anterion and Pentacam HR, which analyzed 53 to 56 eyes [4,5], and provides sufficient precision for estimating the 95% limits of agreement. The study was approved by the Johns Hopkins University School of Medicine Institutional Review Board and adhered to the tenets of the Declaration of Helsinki.

### 2.2. Biometric Measurements

Eligible eyes underwent corneal tomography using swept-source optical coherence tomography (SS-OCT) via the Anterion Cornea App (Heidelberg, Germany) and Scheimpflug tomography via the Pentacam HR (Oculus, Wetzlar, Germany). The Pentacam HR uses a rotating Scheimpflug camera that captures images across 180 degrees of rotation using a 475-nm blue light-emitting diode, generating a three-dimensional reconstruction of the anterior segment from which anterior and posterior corneal curvature, corneal thickness, and related parameters are derived [2]. The Anterion is a swept-source optical coherence tomography device that uses a 1300-nm wavelength light source at a scan speed of up to 50,000 A-scans per second, with an axial resolution of approximately 10 µm [2]. Using the Cornea App, the device acquires 65 radial B-scan images centered on the corneal vertex to provide high-resolution cross-sectional imaging and anterior and posterior corneal biometry [6]. Measurements were obtained by experienced ophthalmic technicians who were trained on and well-versed in using both devices. For each patient, the same technician obtained measurements from both devices. Both devices were located in the same examination room, and all scans were obtained during a single clinic visit on the same day, with patients imaged sequentially over a brief interval. No predetermined interval between scans was specified, the exact time interval between scans was not recorded, and the order of image acquisition was not standardized, reflecting routine clinical workflow. Technicians were not masked to prior measurements, as they uploaded each scan to the institutional imaging platform. Scans with warning signs for poor image quality were excluded: for the Pentacam HR, scans flagged with yellow or red quality indicators denoting fixation, alignment, or blinking errors; and for the Anterion, scans flagged with a warning for low signal strength or ambiguous peaks. Tomographic measurements obtained via each device included anterior keratometry (K1, K2, difference in [Δ] K, average K, and K_max_) in diopters (D); posterior keratometry (K1, K2, ΔK, and average K) in diopters; corneal pachymetry including central corneal thickness (CCT) and the thinnest pachymetric point (TP) in microns (μm); and white-to-white (WTW) in millimeters (mm).

We also recorded patient-level data, including age, sex, race, and ethnicity, as well as eye-level data, including laterality, visual acuity (VA), intraocular pressure, and lens status. VA was recorded with spectacle correction; uncorrected VA was used if corrected VA was unavailable. This study evaluated inter-device agreement between the Anterion and Pentacam HR using a single scan per device for each eye. Within-device repeatability was not assessed, as repeated measurements were not available in this retrospective dataset.

### 2.3. Data Collection and Statistical Analysis

Biometric and clinical data from eligible eyes were retrieved from each imaging device and entered into a REDCap database [7,8]. Statistical analyses were conducted using Stata version 17.0 BE (StataCorp, College Station, TX, USA). Eyes missing a value from either device for a given parameter were excluded from that parameter’s analysis. We assessed agreement between Anterion and Pentacam HR using the Pearson correlation coefficient (ρ), Lin’s concordance correlation coefficient (CCC) with its bias correction factor (Cb), and Bland–Altman (BA) analysis [9,10,11]. The strength of the linear relationship was interpreted as follows: ρ values ≥ 0.8 were considered very strong, 0.6 to <0.8 moderately strong, 0.3 to 0.5 fair and <0.3 poor [12]. CCC values were interpreted according to the benchmarks described by Partik et al. (values >0.95 excellent, >0.90 very good, >0.80 fairly good, >0.70 middling/satisfactory, >0.60 mediocre, >0.50 poor, <0.50 unacceptable) [13]. The bias correction factor measures the degree to which the best-fit line deviates from the line of identity, with Cb = 1 indicating no deviation. BA plots display the mean difference between devices (Anterion—Pentacam HR) against the average of the two devices, with 95% limits of agreement (LoA). When creating BA plots, we checked for proportional bias by regressing the difference on the average of the two measurements and for heteroscedasticity by regressing the absolute residuals on the average. When neither was significant, conventional limits of agreement were used (mean difference ± 1.96 standard deviation [SD]). When proportional bias, heteroscedasticity, or both were present, we derived the limits of agreement using the regression-based BA method [10,11]. To account for the correlation between fellow eyes of the same patient, 95% percentile-based confidence intervals for the bias and LoA were obtained using a cluster bootstrap with 1000 resamples, resampling at the patient level. Mean differences between devices were estimated using generalized estimating equations with the patient as the clustering unit and an independent working correlation structure, since an exchangeable structure model did not converge owing to the near-perfect within-eye correlation of the paired measurements. A Robust (sandwich) variance estimator was used, yielding consistent standard errors that account for within-patient correlation even under a misspecified working correlation [14].

To address the directional nature of astigmatism, we performed a vectorial analysis of anterior and posterior corneal astigmatism for each device. Astigmatism magnitude (ΔK) and its steep meridian were decomposed into Jackson cross-cylinder power-vector components, J0 = (ΔK/2)·cos(2α) and J45 = (ΔK/2)·sin(2α), using the positive-cylinder (steep-meridian) convention [15,16]. For each device and corneal surface, the summated vector mean (SVM) was obtained by averaging the J0 and J45 components across eyes and converting the mean vector back to dioptric magnitude and meridian form, with magnitude = 2·√(J0^2^ + J45^2^) and meridian = ½·tan^−1^ (J45/J0) [16]. In the power-vector convention, the astigmatism meridian is doubled in the forward transform so that opposite meridians represent the same astigmatism; the meridian is therefore recovered by halving the angle, and the factor of 2 in the magnitude formula reverses the ΔK/2 scaling applied when the components are defined. Anterior and posterior astigmatism were displayed for both devices as single-angle polar plots, with each eye’s astigmatism plotted by magnitude (radius) and steep meridian (angle) and the SVM superimposed [17]. Agreement between devices for astigmatism magnitude and for the J0 and J45 components was assessed using the same methods described above.

*p* values less than 0.05 and confidence intervals not overlapping the null value of zero were considered statistically significant.

## 3. Results

Table 1 presents summary characteristics of the study population. We obtained data from 64 eyes of 41 patients. The median patient age was 70.00 years and 73.17% of patients (*N* = 30) were female. Patients were predominantly White (*N* = 25, 60.98%) with smaller proportions of Black (*N* = 6, 14.63%), Asian (*N* = 6, 14.63%), and unknown race (*N* = 4, 9.76%). One patient identified as Hispanic (2.44%). Of the 41 patients, 23 contributed both eyes and 18 contributed one eye, for a total of 64 eyes. Median distance VA was 0.18 logMAR, corresponding to the 20/30 to 20/40 Snellen acuity range, and 78.13% (*N* = 50) of eyes had Snellen VA of 20/40 or better. The majority of eyes had some degree of cataract (*N* = 58, 90.62%) and a minority had clear crystalline lenses (*N* = 4, 6.25%) or were pseudophakic (*N* = 2, 3.12%).

### 3.1. Anterior Keratometry

The linear correlation between Anterion and Pentacam HR for anterior keratometry is presented in Supplementary Figure S1. The two devices demonstrated very strong linear correlation for K_max_ (ρ = 0.942), K1 (ρ = 0.974), K2 (ρ = 0.975), average K (ρ = 0.981), and ΔK (ρ = 0.840). Agreement was very good for K_max_ (CCC = 0.929), excellent for K1 (CCC = 0.974), K2 (CCC = 0.975), and average K (CCC = 0.981), and fairly good for ΔK (CCC = 0.840).

Mean anterior keratometric values for both devices and the inter-device differences are presented in Table 2. No statistically significant mean inter-device differences were found between Anterion and Pentacam HR for K1 (mean difference 0.01 D; *p* = 0.775), K2 (−0.003 D; *p* = 0.951), average K (0.01 D; *p* = 0.801), or ΔK (−0.02 D; *p* = 0.693). For K_max_, Pentacam HR measured 0.24 D steeper than Anterion (45.19 ± 1.45 D versus 44.95 ± 1.45 D), and a statistically significant mean inter-device difference (*p* = 0.002) was noted.

**Figure 1:**
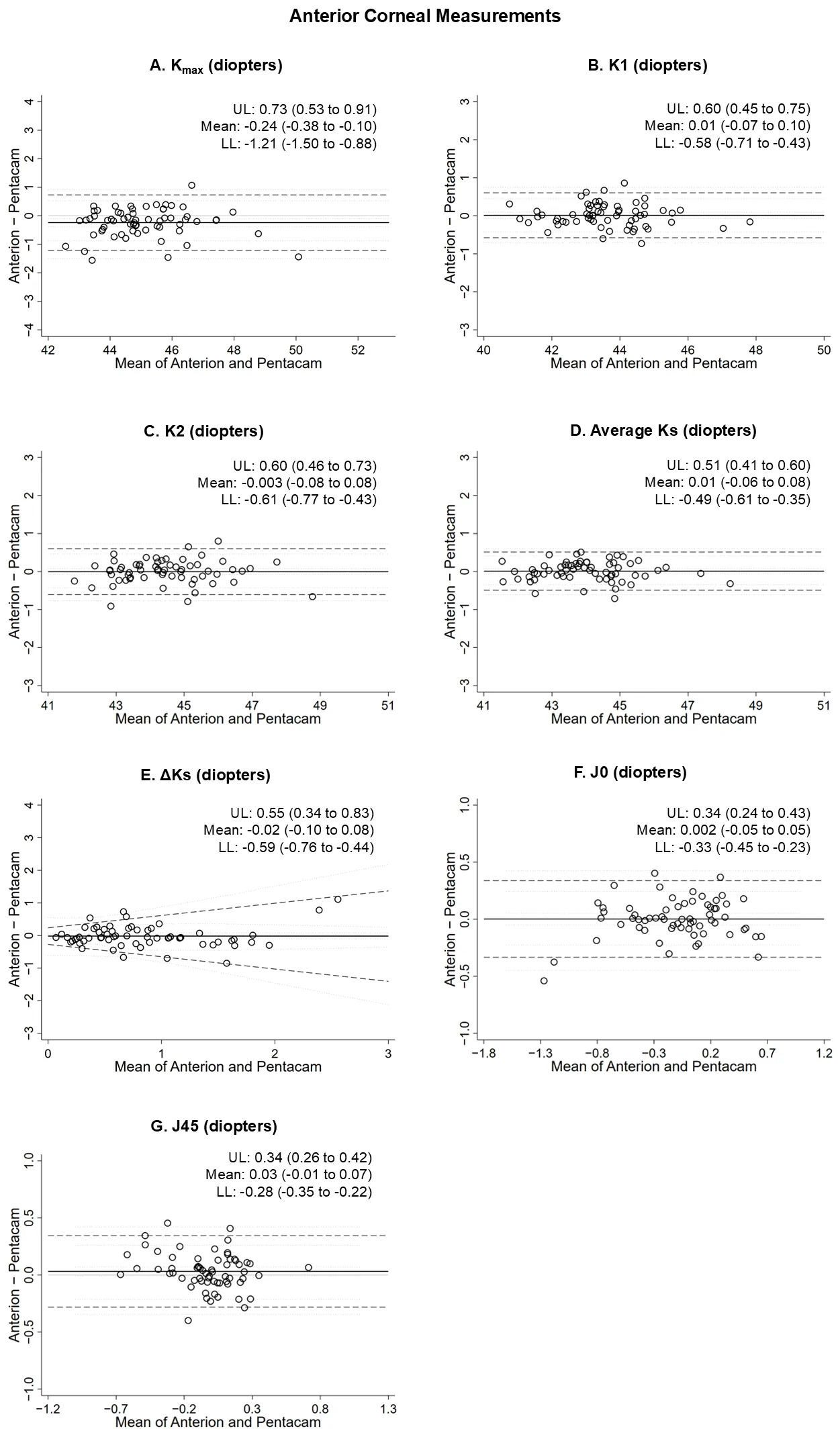
Bland–Altman Plots Comparing Anterior Corneal Tomographic Measurements Obtained by Anterion and Pentacam HR. Legend: Abbreviations: UL = upper limit of agreement; LL = lower limit of agreement; Δ = Difference in Ks. Bland–Altman plots show the difference between the two devices (*y*-axis) plotted against the average of the two devices (*x*-axis). Each open circle represents an individual observation (eye) rather than an individual patient. The solid black line indicates the mean difference (bias) between devices, and the dashed black lines indicate the upper and lower limits of agreement. The solid grey line indicates the line of no difference. The small black dotted lines indicate the 95% percentile-based confidence intervals for the mean bias and limits of agreement, estimated using a clustered bootstrap to account for multiple observations per patient. The 95% confidence intervals are presented in parentheses.

Bland–Altman plots for all anterior parameters are shown in Figure 1. For K1, K2, and average K, the biases noted above were near zero and remained constant across the measurement range, with narrow limits of agreement centered near zero and no proportional bias or heteroscedasticity (all *p* > 0.05) (LoA: K1 −0.58 to 0.60 D; K2 −0.61 to 0.60 D; average K −0.49 to 0.51 D). K_max_ showed a small, fixed bias with wider limits (LoA −1.21 to 0.73 D) that was consistent across the range, without proportional bias (*p* = 0.967) or heteroscedasticity (*p* = 0.546). ΔK demonstrated borderline heteroscedasticity (*p* = 0.048), with the limits of agreement (−0.59 to 0.55 D) widening as mean astigmatism increased, although no proportional bias was present (*p* = 0.753).

### 3.2. Posterior Keratometry

The linear correlation between Anterion and Pentacam HR for posterior keratometry is shown in Supplementary Figure S2. The two devices demonstrated very strong linear correlation for K1 (ρ = 0.974), K2 (ρ = 0.979), average K (ρ = 0.974), and ΔK (ρ = 0.860). Concordance was very good for K2 (CCC = 0.905) and fairly good for K1 (CCC = 0.847), average K (CCC = 0.878), and ΔK (CCC = 0.843).

**Figure 2:**
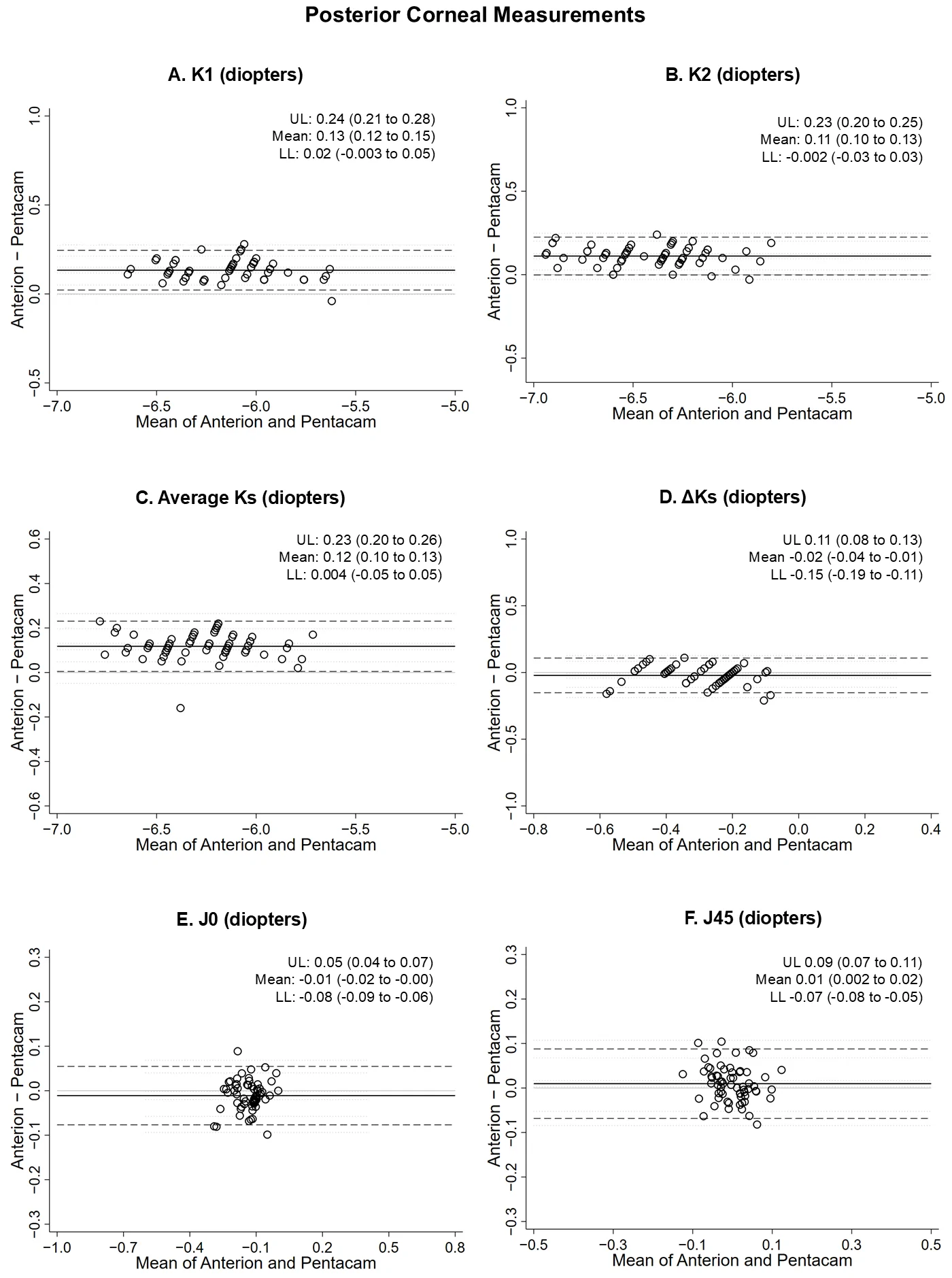
Bland–Altman Plots Comparing Posterior Corneal Tomographic Measurements Obtained by Anterion and Pentacam HR. Legend: Abbreviations: UL = upper limit of agreement; LL = lower limit of agreement; Δ = difference in Ks. Plotting conventions are identical to Figure 1.

Bland–Altman plots for all posterior parameters are shown in Figure 2. Across every parameter, the systematic differences noted above were constant across the measurement range, with stable, parallel limits of agreement and no significant proportional bias or heteroscedasticity (all *p* > 0.05). For K1, K2, and average K, these positive biases are reflected in the predominance of observations above the line of no difference, indicating that Anterion consistently measured slightly less negative values than Pentacam HR (LoA: K1 0.02 to 0.24 D; K2 −0.002 to 0.23 D; average K 0.004 to 0.23 D). Posterior ΔK showed a mean difference near zero with similarly narrow limits (ΔK: −0.15 to 0.11 D).

### 3.3. Vectorial Astigmatism Analysis

**Figure 3:**
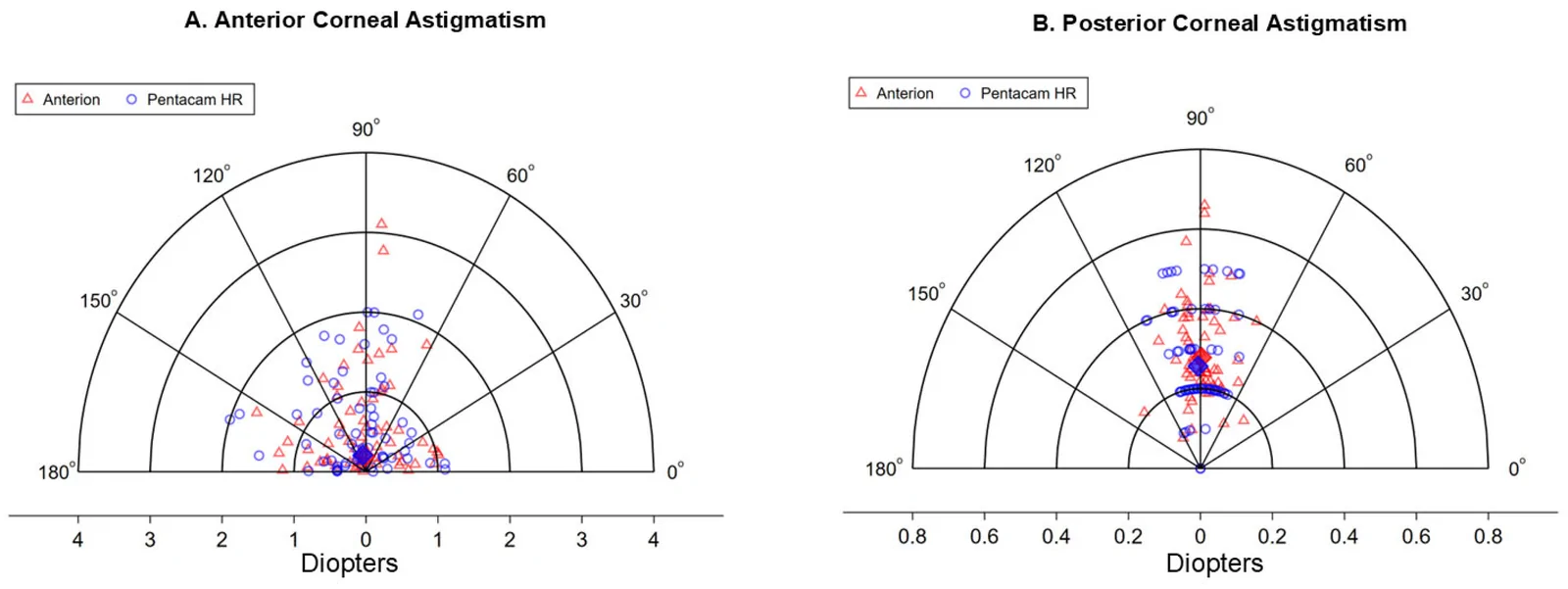
Single-Angle Polar Plots of Anterior and Posterior Corneal Astigmatism Measured by Anterion and Pentacam HR. Legend: Horizontal scale (radius) represents diopters. Each point represents the corneal astigmatism of an individual eye, plotted by magnitude (radial distance, diopters) and steep meridian (polar angle, 0–180°); red triangles denote Anterion and blue open circles denote Pentacam HR. The large diamonds indicate the summated vector mean for each device (Anterion red, Pentacam HR blue), representing the vector average of astigmatism magnitude and orientation across all eyes.

For anterior astigmatism, concordance between devices was very good for J_0_ (ρ = 0.921; CCC = 0.920) and fairly good for J_45_ (ρ = 0.821; CCC = 0.808) (Supplementary Figure S1). No statistically significant mean inter-device differences were found for J0 (mean difference 0.002 D; *p* = 0.936; LoA −0.33 to 0.34 D) or J45 (0.03 D; *p* = 0.129; LoA −0.28 to 0.34 D), consistent with the near-zero, nonsignificant difference in vector magnitude ΔK reported above (Table 2, Figure 1). The SVMs were closely aligned between devices (Anterion 0.19 D at 94°; Pentacam HR 0.21 D at 102°), consistent with the overlapping point distributions and near-coincident SVM vectors seen in Figure 3A.

For posterior astigmatism, concordance was fairly good for J_0_ (ρ = 0.864; CCC = 0.850) and satisfactory for J_45_ (ρ = 0.724; CCC = 0.704) (Supplementary Figure S2). Small but statistically significant mean inter-device differences were detected for J0 (−0.01 D; *p* = 0.018; LoA −0.08 to 0.05 D) and J45 (0.01 D; *p* = 0.007; LoA −0.07 to 0.09 D), paralleling the significant ΔK difference reported above (Table 2; Figure 2). The posterior SVMs remained closely aligned (Anterion 0.28 D at 89°; Pentacam HR 0.26 D at 91°; Figure 3B).

### 3.4. Pachymetry and White-to-White

White-to-white measurements were available for 55 of 64 eyes; the Pentacam HR did not return a WTW value in 9 eyes, whereas the Anterion provided WTW for all eyes. Consistent with our complete-case approach, these 9 eyes were excluded from the WTW analysis only. Compared with included eyes, excluded eyes were from younger patients (median 50 years vs. 72 years), more frequently had a clear crystalline lens (33% vs. 2%), and had slightly lower posterior ΔK measured by Pentacam HR (0.19 ± 0.17 vs. 0.29 ± 0.12 D); the groups did not otherwise differ in sex, race, visual acuity, intraocular pressure, laterality, or any other corneal parameter on either device (Supplementary Table S1). The linear agreement for central corneal thickness (CCT), thinnest location (TP), and white-to-white (WTW) is shown in Supplementary Figure S3. The two devices demonstrated very strong linear correlation for CCT (ρ = 0.974), TP (ρ = 0.981), and WTW (ρ = 0.913). Concordance was excellent for CCT (CCC = 0.951) and TP (CCC = 0.953) and was fairly good for WTW (CCC = 0.890).

**Figure 4:**
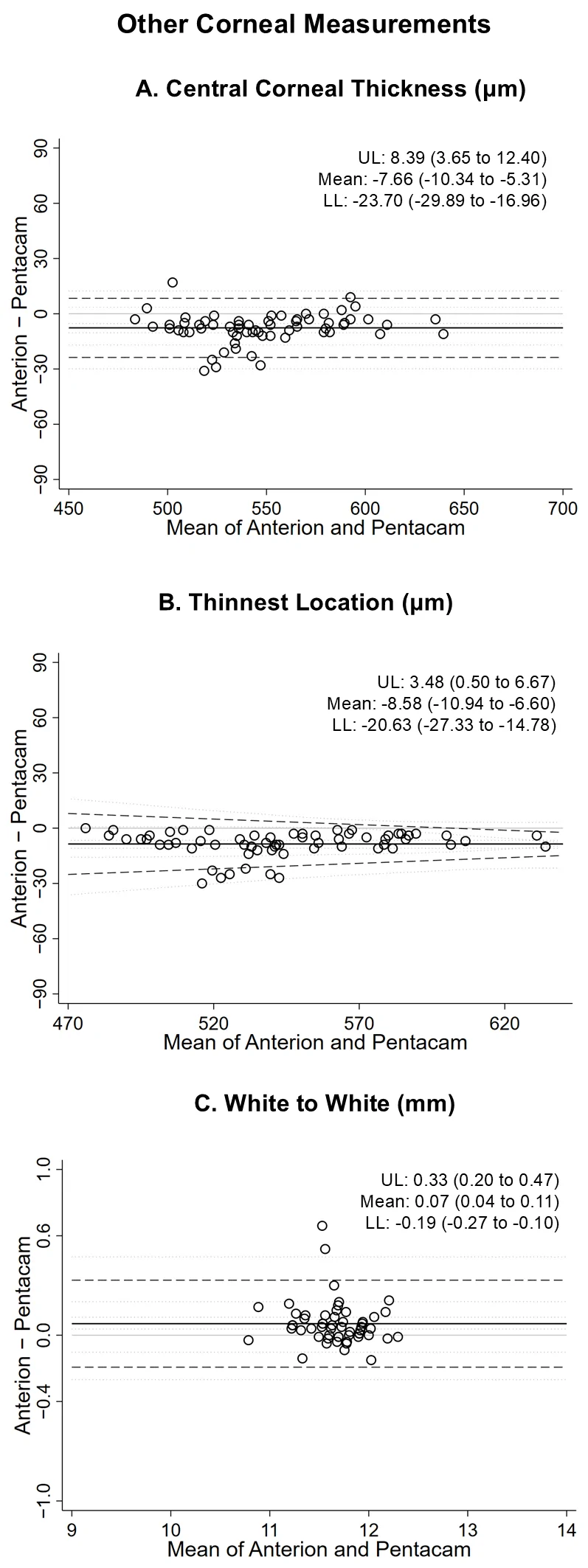
Bland–Altman Plots Comparing Other Corneal Tomographic Measurements Obtained by Anterion and Pentacam HR. Legend: Abbreviations: μm = microns; mm = millimeters; UL = upper limit of agreement; LL = lower limit of agreement. Plotting conventions are identical to Figure 1.

Bland–Altman plots are shown in Figure 4. Anterion systematically measured lower corneal thickness values than Pentacam HR for both CCT and TP. For CCT, the limits of agreement remained approximately parallel across the measurement range (LoA: −23.70 to 8.39 μm) and indicated no significant heteroscedasticity or proportional bias (*p* > 0.05). For TP, no proportional bias was observed (*p* > 0.05) but heteroscedasticity (*p* = 0.03) was present. The limits of agreement narrowed as corneal thickness increased, indicating that inter-device agreement varied across the measurement range (LoA: −20.63 to 3.48 μm). For WTW, Anterion measured slightly higher values than Pentacam HR, with the bias and limits of agreement remaining stable across the measurement range (LoA: −0.19 to 0.33 mm).

## 4. Discussion

In this measurement concordance study comparing Anterion and Pentacam HR, the two devices showed strong concordance for most corneal parameters. We noted no statistically significant mean differences for most anterior keratometry measurements (K1, K2, average K, ΔK, J0, and J45). However, systematic inter-device differences were observed for Kmax, posterior keratometry, pachymetry, and WTW. Although these mean differences were generally small, Bland–Altman analysis demonstrated that the magnitude of the individual-level disagreement varied by parameter, with wider limits of agreement for Kmax and pachymetry. Overall, these findings suggest strong measurement agreement between Anterion and Pentacam HR for many corneal parameters, but their interchangeability should be considered on a parameter-specific basis, particularly when small inter-device differences may influence screening of borderline cases or longitudinal assessments.

### 4.1. Anterior Keratometry

Our findings are similar to those of Ang et al. [3], who found excellent agreement in anterior keratometry between Anterion and the Pentacam AXL Wave Scheimpflug tomographer. However, Ang et al. also observed statistically significant mean differences for anterior K1 (−0.15 D) and K2 (−0.13 D) between the two devices, with Anterion measuring flatter anterior corneal curvature than Pentacam AXL [3]. Several factors may account for this difference. Most notably, Ang et al. compared the Anterion against the Pentacam AXL Wave rather than the Pentacam HR; although both use Scheimpflug tomography, the two platforms differ in their additional modules, software, and processing, which could produce small systematic differences in anterior keratometry [3]. Differences in study populations and in scan acquisition conditions, including operator and fixation, may also have contributed. Perez-Bartolome et al. [5] also found statistically significant differences for anterior keratometry, but the absolute differences between these measurements were so small that they were not clinically relevant. We found that Anterion and Pentacam HR obtained nearly identical measurements for most anterior keratometry values (K1, K2, ΔK, average K). However, the 95% limits of agreement indicate that inter-device differences in an individual eye could approach 0.49–0.61 D. Herber et al. used ±0.50 D as a clinically acceptable agreement margin for keratometry measurements [18]. Although the mean inter-device biases in our study were near zero, the 95% limits of agreement approached or exceeded Herber et al.’s previously published benchmark [18].

Although Anterion and Pentacam HR demonstrated strong concordance and negligible mean differences for most anterior keratometry measurements, small inter-device differences may become clinically relevant in specific surgical settings. For example, differences in ΔK may influence decisions to implant toric IOLs in patients with preoperative astigmatism near 0.75 D, which represents the lower range at which toric correction is considered [19,20]. Clinicians should also use caution when interchanging between devices in eyes with uncommon biometric measurements. In eyes with an axial length < 22 mm, refractive prediction errors greater than ±1.0 D have been reported in 20% of cases, while the refractive prediction errors in eyes with longer axial lengths (>26 mm) have been shown to vary substantially according to the formula used [21,22]. Refractive prediction accuracy also varies in eyes with unusually flat (<42 D) or steep (>46 D) corneas [23,24]. Future studies should determine whether the small inter-device differences observed between Anterion and Pentacam HR meaningfully alter IOL selection or postoperative refractive outcomes in eyes near surgical decision thresholds or with uncommon ocular biometry.

Three eyes in our study had regular with-the-rule anterior astigmatism >2.0 D and showed no evidence of ectasia. Among these, Anterion measured higher anterior ΔK in two eyes, while Pentacam HR measured a higher anterior ΔK in one eye. Future research should evaluate whether measurement concordance between Anterion and Pentacam HR is worse in eyes with high regular astigmatism or corneal ectasia, as this could have implications for refractive surgery planning and ectasia screening.

The only anterior keratometry parameter with a statistically significant mean inter-device measurements was K_max_, for which Anterion obtained mean values 0.24 D flatter than Pentacam HR. Although K_max_ is an important biomarker for detecting keratoconus and monitoring its progression, the magnitude of the mean inter-device difference observed in our study may have limited clinical relevance [25,26]. Herber et al. reported coefficients of repeatability for K_max_ of 0.37 D with Pentacam HR and 0.36 D with Anterion in normal eyes, indicating that the 0.24-D mean inter-device difference observed in our cohort is within the reported test–retest variability of both devices [18]. Furthermore, an increase in K_max_ of approximately 1.0 D over 6–12 months has commonly been used as a criterion for clinically significant keratoconus progression [27,28,29]. Therefore, the 0.24-D mean difference observed in our study is small relative to changes commonly used to identify clinically meaningful ectatic progression.

However, smaller systematic differences may be more relevant in borderline cases. Heidari et al. found that K_max_ was substantially less effective at distinguishing normal from subclinical keratoconus (AUC = 0.619) than normal from clinical keratoconus (AUC = 0.991) [30]. Similarly, Randleman et al. found no significant difference in K_max_ between normal and subclinical keratoconus eyes, with K_max_ demonstrating limited ability to distinguish between the two groups (AUROC = 0.56) [31]. Therefore, a systematic inter-device difference of 0.24 D is less likely to alter clinical interpretation in established keratoconus but may add uncertainty when distinguishing normal from subclinical disease. This consideration is also relevant to longitudinal monitoring because K_max_ repeatability worsens with increasing keratoconus severity. Herber et al. reported repeatability coefficients of 0.62 D and 1.33 D for Pentacam HR and 0.50 D and 0.78 D for Anterion in mild and moderate keratoconus, respectively [18]. Clinicians should use caution when using multiple devices for longitudinal monitoring, particularly when small changes in K_max_ may influence assessment of progression during earlier stages of disease. Further research is needed to determine the repeatability and measurement concordance of Anterion and Pentacam HR among keratoconus patients and evaluate the clinical significance of any differences.

### 4.2. Posterior Keratometry

Compared with Pentacam HR, Anterion measured 0.13 D, 0.11 D, and 0.12 D flatter measurements for posterior K1, K2, and average K, respectively. The 95% limits of agreement did not extend farther than 0.24 D. The Global Consensus on Keratoconus and Ectatic disease recognizes posterior corneal steepening as an important marker of ectatic progression in keratoconus [32]. In eyes with keratoconus, Romano et al. reported Pentacam HR repeatability limits of 0.23–0.25 D, 0.24–0.33 D, and 0.18–0.24 D for posterior K1, K2, and average K, respectively [33]. Although the mean inter-device differences in our study were smaller than these reported repeatability limits, the 95% limits of agreement approached the magnitude of measurement variability for Pentacam HR. Heidari et al. found nearly identical posterior K2 measurements in normal (−6.52 D) versus subclinical keratoconus eyes (−6.53 D), whereas posterior K2 was substantially steeper in eyes with established keratoconus (−7.37 D) [30]. These findings suggest that small inter-device differences in posterior K2 may be more relevant in subclinical cases rather than eyes with established disease. Nicula et al. demonstrated that using a −6.95 D cutoff for distinguishing clinical from subclinical keratoconus had an AUC of 0.852 with 62.9% sensitivity and 97.4% specificity [34]. Although these findings are population-specific, they further demonstrate that posterior K2 can distinguish between different disease stages, while emphasizing that systematic inter-device differences may be more relevant in eyes near diagnostic boundaries. Clinicians should be cautious when switching between the two devices during longitudinal keratoconus monitoring given that small changes in posterior curvature could influence assessment of progression in early disease.

Our findings are consistent with three other studies [3,4,5] which showed that Anterion measures flatter posterior keratometry than Pentacam HR. Device-specific differences in imaging technology may contribute to the systematic offsets observed. This difference may be because Pentacam HR utilizes blue light at a wavelength of 475 nm, whereas Anterion uses 1300 nm swept source OCT, leading to differences in corneal segmentation, sampling, and reconstruction between the two devices [6]. Posterior corneal measurements may contribute to total corneal power and astigmatism calculations used in cataract surgery planning. Since Anterion and Pentacam HR measure nearly identical anterior keratometry, the systematic differences in posterior keratometry measurements may propagate to differences in calculated total corneal power [22]. However, our study did not evaluate total keratometry-based IOL calculations; therefore, we cannot determine whether the observed 0.12 D difference in posterior average K would alter IOL selection. More research is needed to determine whether Anterion and Pentacam HR may be considered interchangeable by cataract surgeons incorporating posterior keratometry into IOL calculations.

### 4.3. Vectorial Astigmatism Analysis

To address the directional nature of astigmatism, which scalar comparisons cannot capture, we supplemented our analysis with a power-vector approach. Anterior ΔK, J0, and J45 showed no significant mean inter-device differences, with closely aligned summated vector means, demonstrating similar group-level estimates of the magnitude and direction of astigmatism. Although posterior J0 and J45 differences reached statistical significance, the mean differences were only 0.01 D, with 95% limits of agreement extending no farther than 0.09 D from zero. Posterior J45 demonstrated lower concordance (CCC = 0.704) than J0, which was consistent with Gim et al., who found posterior J45 to be the least concordant vector component when comparing Anterion and Pentacam HR [4]. This may partly reflect the mathematical dependence of J45 on the astigmatic axis. According to the power-vector formulation, J45 is proportional to the sine of twice the astigmatic axis [16]. Therefore, when astigmatism is oriented near the horizontal or vertical meridians, J45 approaches zero, and small inter-device differences in measured axis can change the magnitude or sign of J45 despite minimal differences in the underlying astigmatism [16]. Differences in posterior surface imaging and reconstruction between SS-OCT and Scheimpflug technologies may further contribute to this variability [6].

The polar plots showed substantial overlap between devices, with closely aligned summated vector means for both anterior and posterior astigmatism. This is essential for determining patient eligibility for toric IOL implantation, selecting the axis of toric IOL alignment, or the meridian of surgical incision placement. Furthermore, accurate assessment of posterior corneal astigmatism is clinically relevant for toric IOL planning because accounting for posterior corneal curvature improves postoperative residual astigmatism [35]. Although the posterior vector differences observed in our study were small, we did not evaluate their effects on toric IOL selection or postoperative residual astigmatism. Future studies should determine whether these inter-device differences are clinically relevant for astigmatism correction during cataract surgery planning.

### 4.4. Pachymetry and White-to-White

Anterion measured CCT and TP 7.66 μm and 8.58 μm thinner than Pentacam HR, respectively. Herber et al. defined ±10 μm as a clinically acceptable agreement margin for corneal thickness when comparing two devices [18]. Although the mean inter-device differences in our study were within this study-defined margin, the 95% limits of agreement extended from −23.70 to +8.39 μm for CCT and −20.63 to +3.48 μm for TP, indicating greater disagreement in some individual eyes. Refractive surgery screening generally considers pachymetry in relation to predicted residual stromal bed and percent tissue altered, with increased ectasia risk reported for residual stromal bed values below approximately 250–300 µm and percent tissue altered ≥40% [36,37,38]. Although the average pachymetric differences we observed between the two devices were modest, individual differences exceeding 20 µm may be relevant in eyes near refractive surgery treatment thresholds. Our findings are consistent with Perez-Bartolome et al., [5] who reported that Anterion measured a mean CCT that was 5.76 μm thinner than that measured by Pentacam HR. Differences in tissue penetration, corneal segmentation, and measurement reference locations may contribute to the observed systematic pachymetric offsets.

Horizontal WTW is useful for characterizing anterior segment anatomy and has clinical relevance in assessing microcornea or megalocornea, phakic IOL planning, and contact lens fitting [39,40]. Since implantable collamer lens (ICL) sizes are selected in 0.50 mm increments, WTW differences ≥ 0.50 mm have been suggested as clinically significant for ICL sizing [41]. In our study, the mean inter-device WTW difference was 0.07mm and the 95% limits of agreement ranged from −0.19 to 0.33 mm, suggesting that observed WTW differences are unlikely to affect ICL surgical planning.

### 4.5. Strengths and Limitations

Strengths of this study include the detailed analysis of all clinically relevant corneal measurements obtained by both the Anterion Cornea App and Pentacam HR, including posterior keratometry, pachymetry, and white-to-white diameter within a single cohort and a uniform analytic framework. In addition, our data were drawn from a US clinical population, whereas prior device comparisons were performed in single non-US centers. Prior studies did not evaluate the full set of parameters assessed here, particularly pachymetry or white-to-white diameter [3,4]. Our study analyzed additional corneal measurements such as posterior keratometry, astigmatism (ΔK), and pachymetry as well as anterior keratometry. Examining all corneal parameters together provides a more comprehensive assessment of the strengths and limitations of the two devices. For example, differences across three separate corneal measurements (anterior K_max_, posterior Ks, and thinnest pachymetry) all indicate that Anterion could hypothetically be more specific but less sensitive than Pentacam HR for detection of corneal ectasia without the presence of additional screening tools such as epithelial thickness mapping or the recently FDA-approved SCORE algorithm [42,43].

This study has limitations. Our analysis was limited to healthy eyes with no clinically apparent corneal disease. Further studies are warranted to analyze differences in measurements obtained by Anterion and Pentacam HR in patients with corneal pathology such as keratoconus, corneal edema, corneal endothelial disease, or irregular astigmatism. Corneal normality was defined by documented clinical diagnosis rather than by tomographic screening indices; subclinical or early ectatic change may therefore not have been detected, although all eyes were examined by an ophthalmologist and none had documented pathological findings. Our sample was modest in size (64 eyes of 41 patients) and drawn from a single academic institution and was predominantly older, female, and affected by cataract. Our findings should therefore be interpreted as reflecting a clinic-based population undergoing evaluation rather than the general US population. Another limitation is that a single technician did not obtain scans for all patients. However, all technicians were highly trained and experienced in the use of both devices, including the newer Anterion Cornea App, which has a relatively short learning curve. The scan acquisition order was not standardized or documented. Both eyes of many patients were included, and although we accounted for this correlation using patient-level clustering, the non-independence of fellow eyes remains a design limitation. We did not perform test–retest repeatability or external validation, which future studies should address. To ensure image quality, all scans included in this analysis met image quality thresholds, and scans flagged as poor quality due to image-capture errors or low signal strength were excluded from the study. Future studies should evaluate the imageability, image quality, and error rate of Anterion and Pentacam HR since these factors are clinically relevant in patients with ocular comorbidities, poor cooperation, or difficulty positioning for imaging.

In summary, Anterion and Pentacam HR showed good to excellent measurement concordance and negligible mean inter-device differences for most anterior keratometry and astigmatism parameters. Small systematic differences were observed for K_max_, posterior keratometry, pachymetry, and WTW. These small differences may be relevant in borderline cases or during longitudinal disease monitoring. Therefore, the interchangeability between Anterion and Pentacam HR should be considered on a parameter-specific basis.

## Figures and Tables

**Table 1:** Demographics and Clinical Characteristics of Study Participants.

Characteristics	Values
*N*, eyes	64
*N*, patients	41
Age, median (IQR)	70.00 (65.00, 76.00)
Laterality, *N* (%)	
Right eye	30 (46.88)
Left eye	34 (53.12)
Sex, *N* (%)	
Female	30 (73.17)
Male	11 (26.83)
Race, *N* (%)	
White	25 (60.98)
Black	6 (14.63)
Asian	6 (14.63)
Unknown	4 (9.76)
Hispanic ethnicity, *N* (%)	1 (2.44)
IOP, median (IQR)	14.00 (12.00, 16.50)
Snellen visual acuity, *N* (%)	
20/20 or better	11 (17.19)
>20/20 to ≤20/40	39 (60.94)
>20/40 to ≤20/80	9 (14.06)
20/80 or worse	3 (4.69)
Missing	2 (3.12)
LogMAR visual acuity, median (IQR)	0.18 (0.10, 0.30)
Lens status, *N* (%)	
Clear crystalline lens	4 (6.25)
Cataract	58 (90.62)
Pseudophakia	2 (3.12)

Abbreviations: IOP = intraocular pressure; IQR = interquartile range; *N* = number.

**Table 2:** Differences in Corneal Tomography Measurements between Anterion and Pentacam HR.

Measurement Type	Measurement	Anterion, Mean (SD)	Pentacam HR, Mean (SD)	Anterion—Pentacam HR (95% CI)	*p* Value
Anterior corneal curvature	K_max_ (diopters)	44.95 (1.45)	45.19 (1.45)	−0.24 (−0.39, −0.09) *	0.002 *
	K1 (diopters)	43.60 (1.31)	43.59 (1.30)	0.01 (−0.07, 0.10)	0.775
	K2 (diopters)	44.43 (1.38)	44.43 (1.37)	−0.003 (−0.08, 0.08)	0.951
	Average K (diopters)	44.01 (1.31)	44.00 (1.32)	0.01 (−0.06, 0.08)	0.801
	ΔK (diopters)	0.83 (0.59)	0.85 (0.57)	−0.02 (−0.11, 0.07)	0.693
	J_0_ (diopters)	−0.09 (0.44)	−0.10 (0.42)	0.002 (−0.05, 0.05)	0.936
	J_45_ (diopters)	−0.01 (0.24)	−0.04 (0.28)	0.03 (−0.01, 0.07)	0.129
Posterior corneal curvature	K1 (diopters)	−6.06 (0.24)	−6.20 (0.25)	0.13 (0.12, 0.15) *	<0.001 *
	K2 (diopters)	−6.36 (0.28)	−6.45 (0.28)	0.11 (0.10, 0.13) *	<0.001 *
	Average K (diopters)	−6.21 (0.25)	−6.33 (0.26)	0.12 (0.10, 0.13) *	<0.001 *
	ΔK (diopters)	−0.30 (0.12)	−0.28 (0.13)	−0.02 (−0.04, −0.00) *	0.018 *
	J_0_ (diopters)	−0.14 (0.07)	−0.13 (0.06)	−0.01 (−0.02, −0.00) *	0.018 *
	J_45_ (diopters)	0.003 (0.05)	−0.01 (0.06)	0.01 (0.003, 0.02) *	0.007 *
Other corneal measurements	Central corneal thickness (μm)	544.59 (36.09)	552.25 (34.91)	−7.66 (−10.16, −5.15) *	<0.001 *
	Thinnest location (μm)	540.06 (36.13)	548.64 (35.34)	−8.58 (−10.79, −6.36) *	<0.001 *
	White-to-white (mm)	11.70 (0.31)	11.63 (0.33)	0.07 (0.03, 0.11) *	<0.001 *

Abbreviations: CI = confidence interval; mm = millimeters; SD = standard deviation; μm = microns; Δ: difference in Ks. Difference in means (Anterion minus Pentacam HR), CI and *p* values are derived from generalized estimating equation models. An asterisk (*) indicates a statistically significant mean inter-device difference. All measurements were obtained in *N* = 64 eyes except for white-to-white (WTW). WTW was available for 55 eyes; the Pentacam HR did not return a WTW value in 9 eyes, which were excluded from the WTW analysis.

## Data Availability

The data that support the findings of this study is publically unavailable due to privacy or ethical restriction and can be made available from the corresponding author upon reasonable request.

## Supplementary Materials

The following supporting information can be downloaded at: https://www.mdpi.com/article/10.3390/bioengineering13080944/s1, Supplementary Figure S1: Scatter Plots Comparing Anterior Corneal Tomographic Measurements Obtained by Anterion and Pentacam HR; Supplementary Figure S2: Scatter Plots Comparing Posterior Corneal Tomographic Measurements Obtained by Anterion and Pentacam HR; Supplementary Figure S3: Scatter Plots Comparing Other Corneal Tomographic Measurements Obtained by Anterion and Pentacam HR; Supplementary Table S1: Demographics and Clinical Characteristics of Study Participants for White-to-White analysis.
