# Supplementary material for: Concordance of Corneal Tomographic Measurements Obtained via Swept-Source Optical Coherence Tomography Versus Scheimpflug Tomography
Source: Bioengineering (Basel). 2026 Aug 21;13(8):944. doi: 10.3390/bioengineering13080944 (PMC13509365; doi:10.3390/bioengineering13080944)
Supplement: Supplementary file 1 [file bioengineering-13-00944-s001.zip › bioengineering-4440386-supplementary.pdf]

Supplementary Table S1: Demographics and Clinical Characteristics of Study Participants for White-to-White analysis.

Characteristic	Included in WTW analysis (N=55 eyes)	Excluded from WTW analysis (N=9 eyes)	P value[a]
Patient and eye characteristics			
Age, years, median (IQR)[b]	72.00 (65.00, 77.00)	50.00 (43.00, 70.00)	0.014
Female sex, N (%)[b]	40 (72.73)	6 (66.67)	0.703
Race, N (%)[bc]			
White	35 (63.64)	3 (33.33)	0.142
Black	6 (10.91)	3 (33.33)	0.106
Asian	9 (16.36)	2 (22.22)	0.646
Other or unknown	6 (10.91)	0 (0)	0.582
Hispanic ethnicity, N (%)[b]	2 (3.64)	0 (0)	1.000
Visual acuity, logMAR, median (IQR)[d]	0.18 (0.10, 0.30)	0.24 (0.05, 0.35)	0.928
Intraocular pressure, mmHg, median (IQR)	14.00 (11.00, 16.00)	15.00 (14.00, 20.00)	0.160
Right eye, N (%)	26 (47.27)	4 (44.44)	1.000
Lens status, N (%)			0.014
Clear crystalline lens	1 (1.82)	3 (33.33)	
Cataract	52 (95.55)	6 (66.67)	
Pseudophakic	2 (3.64)	0 (0)	
Corneal measurements, Pentacam HR [mean (SD)]			
Anterior K1, D	43.61 (1.38)	43.43 (0.99)	0.906
Anterior K2, D	44.46 (1.42)	44.28 (1.08)	0.966
Anterior Kavg, D	44.03 (1.37)	43.83 (0.97)	0.883
Anterior ΔK, D	0.85 (0.57)	0.84 (0.58)	0.912
Anterior Kmax, D	45.24 (1.52)	44.86 (0.89)	0.604
Posterior K1, D	−6.19 (0.25)	−6.24 (0.27)	0.473

Characteristic	Included in WTW analysis (N=55 eyes)	Excluded from WTW analysis (N=9 eyes)	P value[a]
Posterior K2, D	−6.48 (0.28)	−6.46 (0.30)	0.958
Posterior Kavg, D	−6.32 (0.26)	−6.34 (0.27)	0.614
Posterior ΔK, D[e]	0.29 (0.12)	0.19 (0.17)	0.023
CCT, µm	552.64 (34.73)	549.89 (38.03)	0.958
TP, µm	549.13 (35.04)	545.67 (39.18)	0.936
Corneal measurements, Anterion [mean ((SD)]			
Anterior K1, D	43.62 (1.35)	43.48 (1.05)	0.943
Anterior K2, D	44.42 (1.40)	44.50 (1.33)	0.572
Anterior Kavg, D	44.01 (1.35)	43.98 (1.12)	0.817
Anterior ΔK, D	0.80 (0.55)	1.02 (0.83)	0.585
Anterior Kmax, D	44.96 (1.47)	44.89 (1.42)	0.837
Posterior K1, D	−6.06 (0.24)	−6.08 (0.28)	0.951
Posterior K2, D	−6.37 (0.27)	−6.33 (0.29)	0.876
Posterior Kavg, D	−6.21 (0.25)	−6.20 (0.27)	0.936
Posterior ΔK, D[e]	0.31 (0.11)	0.25 (0.17)	0.057
CCT, µm	544.55 (36.39)	544.89 (36.35)	0.996
TP, µm	540.29 (35.91)	538.67 (39.65)	0.996

Legend: Abbreviations: WTW = white-to-white; SD = standard deviation; D = diopters; ΔK = difference between K1 and K2 (astigmatism); Kavg = average keratometry; CCT = central corneal thickness; TP = thinnest pachymetric point; µm = microns.
Nine eyes of 8 patients were excluded from the WTW analysis. In all 9 eyes, the WTW measurement was unavailable from Pentacam HR only; WTW was available from Anterion for all 64 eyes.
[a] Continuous variables were compared using the Wilcoxon rank-sum test (exact p-values reported) and categorical variables using Fisher's exact test. P values are descriptive given the small number of excluded eyes, and tests do not account for correlation between fellow eyes.
[b] Age, sex, race, and ethnicity are patient-level characteristics summarized at the eye level.
[c] Each race category was compared between groups as a binary indicator using Fisher's exact test.
[d] Visual acuity was available for 62 of 64 eyes (54 included, 8 excluded).
[e] Posterior ΔK is reported as an absolute magnitude for both devices.

Supplementary Figure S1: Scatter Plots Comparing Anterior Corneal Tomographic Measurements Obtained by Anterion and Pentacam HR

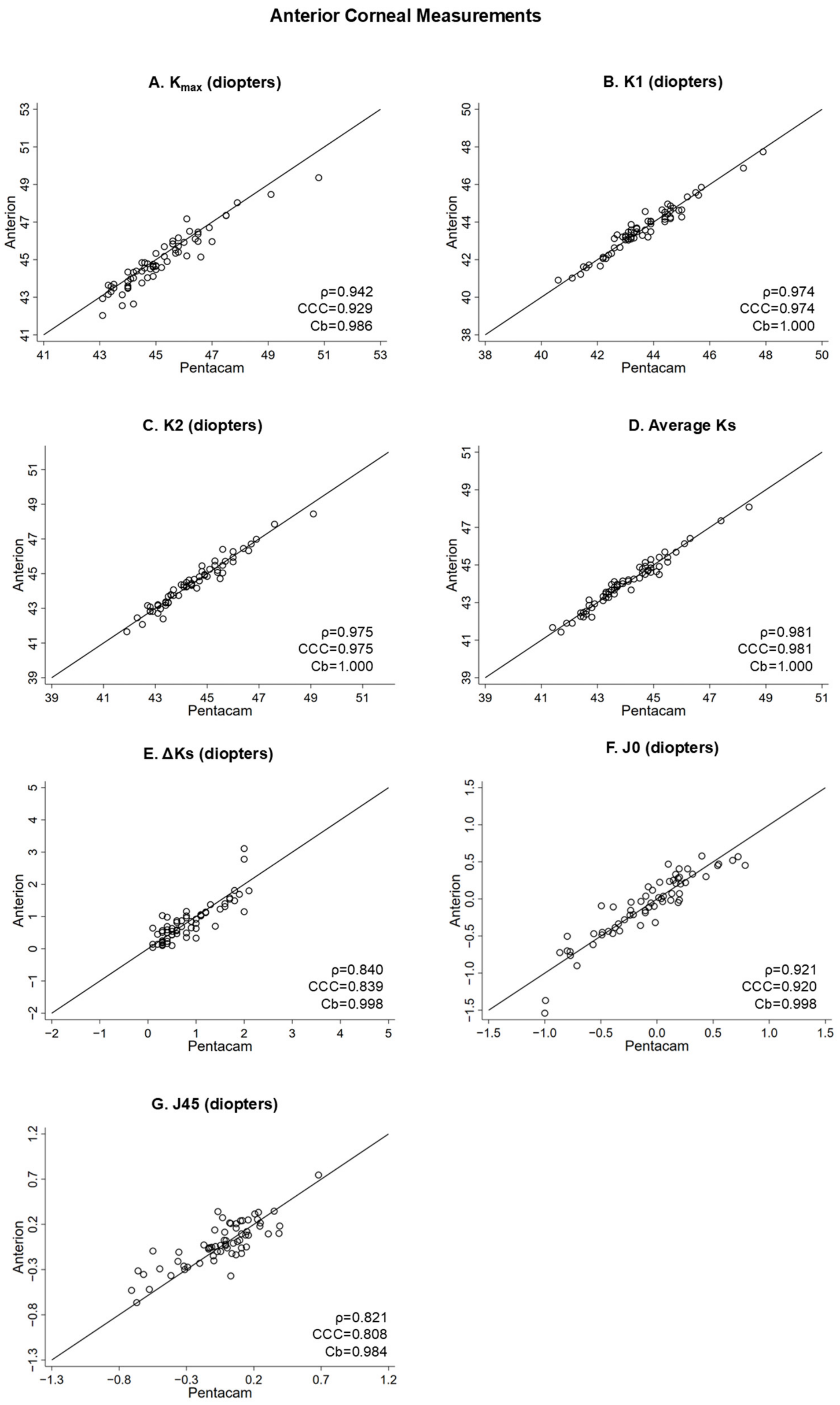

Legend**:** ρ = Pearson correlation coefficient; CCC = Lin's concordance correlation coefficient; Cb = bias correction factor; Δ = Difference in Ks. Each open circle represents an individual observation (eye) rather than an individual patient. The solid line represents the line of perfect agreement between the two devices (x = y).

Supplementary Figure S2: Scatter Plots Comparing Posterior Corneal Tomographic Measurements Obtained by Anterion and Pentacam HR

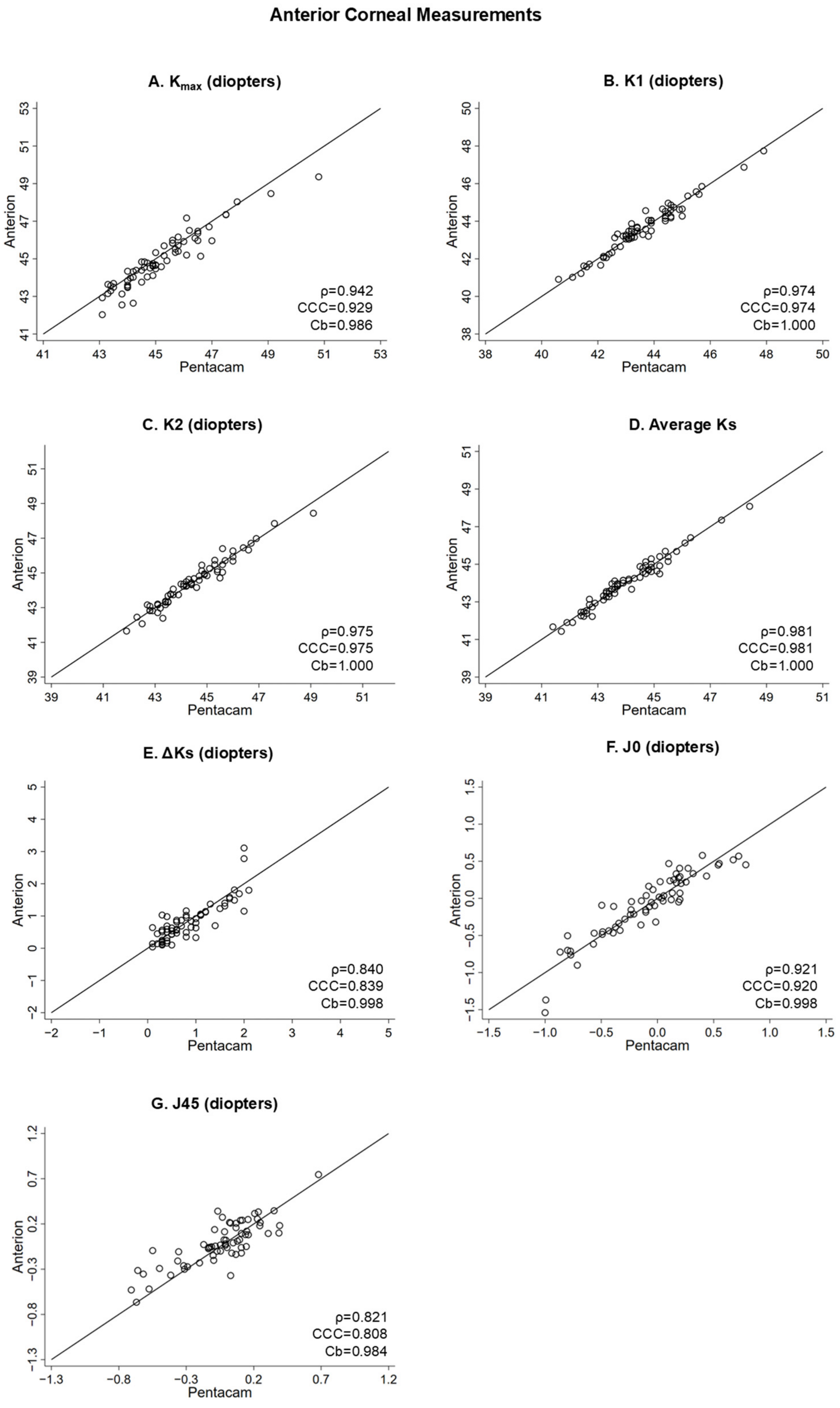

Legend**:** Abbreviations: ρ = Pearson correlation coefficient; CCC = Lin's concordance correlation coefficient; Cb = bias correction factor; Δ = Difference in Ks. Plotting conventions are identical to Supplementary Figure 1.

Supplementary Figure S3: Scatter Plots Comparing Other Corneal Tomographic Measurements Obtained by Anterion and Pentacam HR

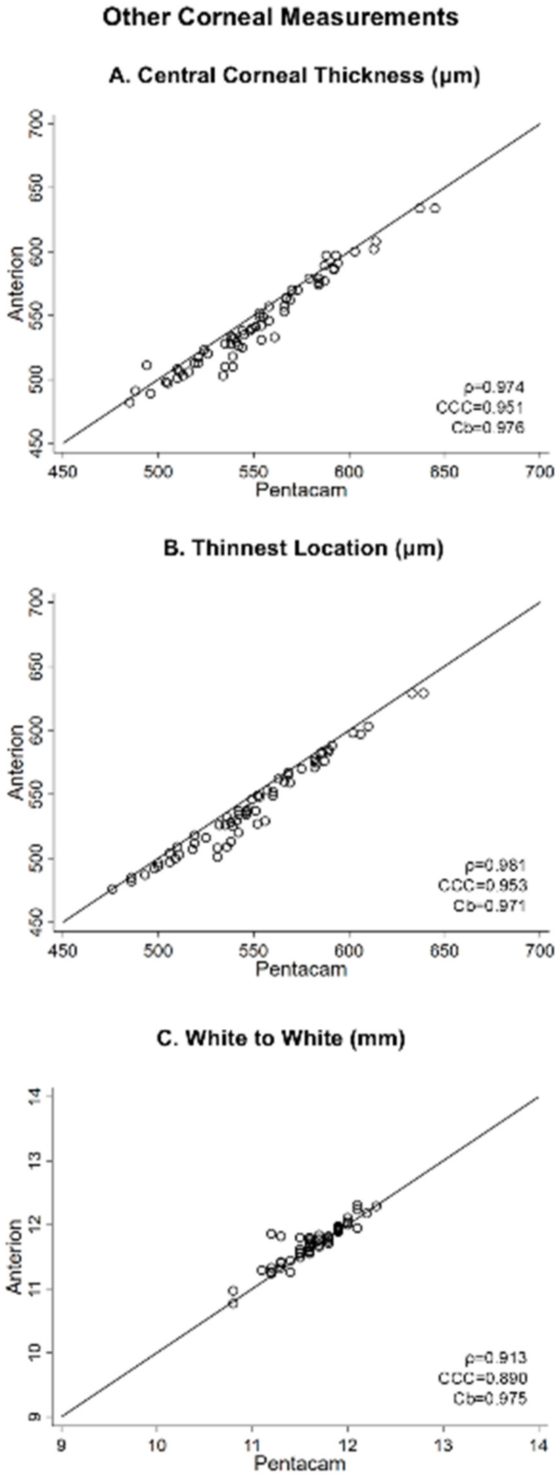

Legend: Abbreviations: μm = microns; mm = millimeters; ρ = Pearson correlation coefficient; CCC = Lin's concordance correlation coefficient; Cb = bias correction factor. Plotting conventions are identical to Supplementary Figure 1.
